# Safe and Efficient Procedures and Training System for Endoscopic Submucosal Dissection

**DOI:** 10.3390/jcm12113692

**Published:** 2023-05-26

**Authors:** Yu Kamitani, Kouichi Nonaka, Yoshitsugu Misumi, Hajime Isomoto

**Affiliations:** 1 Department of Digestive Endoscopy, Tokyo Women’s Medical University Hospital, 8-1 Kawada-cho, Shinjuku-ku, Tokyo 162-8666, Japan; 2 Division of Gastroenterology and Nephrology, Department of Multidisciplinary Internal Medicine, Faculty of Medicine, Tottori University, 36-1 Nishicho, Yonago 683-8504, Japan

**Keywords:** endoscopic submucosal dissection, perforation, hemorrhage

## Abstract

Recent improvements in endoscopists’ skills and technological advances have allowed endoscopic submucosal dissection (ESD) to become a standard treatment in general hospitals. As this treatment entails a high risk of accidental perforation or hemorrhage, therapeutic procedures and training methods that enable ESD to be conducted more safely and efficiently are constantly being developed. This article reviews the therapeutic procedures and training methods used to improve the safety and efficiency of ESD and describes the ESD training system used in a Japanese university hospital at which the number of ESD procedures has gradually increased in a newly established Department of Digestive Endoscopy. During the establishment of this department, the ESD perforation rate was zero among all procedures, including those conducted by trainees.

## 1. Introduction

Recent improvements in endoscopists’ skills and technological advances have allowed endoscopic submucosal dissection (ESD) to become a standard treatment in general hospitals in Western nations and Asia, including Japan [1,2,3,4,5]. However, there is a high risk of perforation or hemorrhage during ESD. Therefore, devices to enable ESD to be conducted more safely and efficiently, and novel training methods, are constantly being developed.

The Department of Digestive Endoscopy at our university hospital in Tokyo was established in April 2020. Trainees were recruited from all areas of Japan for the clinical practice of therapeutic endoscopy in the forms of diagnostic endoscopy and ESD, education, and research. Prior to the establishment of this department, the number ESD procedures each year conducted at our hospital was less than fifty. This number has increased to approximately 400 esophageal, gastric, and colorectal ESD procedures per year, since the establishment of the digestive endoscopy department less than three years ago. During this time, the ESD perforation rate has remained at 0% among all procedures, including those conducted by trainees. This review reports on the methods used to improve the safety and efficacy of ESD at our hospital. The training methods introduced at our hospital are also discussed.

## 2. Establishment of the Digestive Endoscopy Department and Therapeutic Outcomes for ESD

The Department of Digestive Endoscopy at the Tokyo Women’s Medical University Hospital, located in the Shinjuku Ward, was established in April 2020. The department initially included two experts and four trainees. Additional trainees wishing to study ESD at our hospital were recruited from across Japan through a system of student exchanges from other hospitals within the country. As of December 2022, the department includes two experts and six trainees. Prior to the establishment of the Department of Digestive Endoscopy, our hospital conducted less than 50 ESD procedures each year, and the goal of the new department was to conduct 400–500 procedures a year. Therefore, trainees were also involved in establishing the clinical department and treating patients from the beginning, providing them with a unique experience.

Between April 2020 and December 2022, gastric, colorectal, or esophageal ESD was conducted on 818 patients. The indicative lesion was located in the stomach in 346 patients, the colorectum in 361 patients, and the esophagus in 111 patients. A total of 346 patients underwent gastric ESD, including 252 males and 94 females. The median age of patients who underwent gastric ESD was 73 years (range 35–92 years). The median lesion size was 13.5 mm (range 1–100 mm) overall, 14 mm (range 1–100 mm) for experts, and 8 mm (range 1–68 mm) for trainees. The lesion was located in the upper third of the stomach in 63 patients, the middle third in 109 patients, and the lower third in 174 patients. A histopathological diagnosis, based on the guidelines for ESD and endoscopic mucosal resection (EMR) for early gastric cancer [2], or endoscopic findings suggestive of carcinoma were indications for ESD, even if adenoma was diagnosed via preoperative biopsy. Adenoma was diagnosed in 53 patients, carcinoma in 274 patients (the differentiated type in 239 patients and the undifferentiated type in 35 patients), and other lesions in 19 patients. The pathological invasion depth of the tumor was intramucosal (T1a) carcinoma in 232 patients and submucosal invasive (T1b) carcinoma in 41 patients. The median operating time was 35.5 min (range 3–277 min) overall, 30 min (range 3–277 min) for experts, and 38 min (range 3–190 min) for trainees. Of the 346 gastric ESD procedures, 137 were conducted by experts and 209 by trainees. The ESD perforation rate was 0% for both experts and trainees. The complete resection rate was 98.6% (Table 1).

A total of 361 patients underwent colorectal ESD, including 208 males and 153 females. The median patient age was 68 years (range 32–91 years). The median lesion size was 23 mm (range 4–88 mm) overall, 26 mm (range 5–88 mm) for experts, and 20 mm (range 4–67 mm) for trainees. Of the 361 lesions, 268 were located in the colon and 93 in the rectum. The histopathological diagnosis, based on the Japan Gastroenterological Endoscopy Society guidelines for colorectal ESD/EMR [3], was adenoma in 99 patients, intramucosal (Tis) carcinoma in 149 patients, submucosal invasive (T1) carcinoma in 39 patients, sessile serrated lesion (SSL) in 67 patients, and other lesions in 7 patients. SSLs > 20 mm that could not be resected en bloc and lesions with suspected dysplasia or malignancy were treated by ESD. The median operating time was 37 min (range 5–294 min) overall, 36 min (range 5–294 min) for experts, and 38 min (range 8–118 min) for trainees. Of the 361 colorectal ESD procedures, 197 were conducted by experts and 164 by trainees. The ESD perforation rate was 0% for both experts and trainees. The complete resection rate was 98.9% (Table 2).

A total of 111 patients underwent esophageal ESD, including 92 males and 19 females. The median patient age was 71 years (range 50–87 years). The median lesion size was 16 mm (range 4–61 mm) overall, 18 mm (range 4–61 mm) for experts, and 15 mm (range 5–41 mm) for trainees. The lesion was located in the cervical esophagus in 2 patients, upper thoracic esophagus in 12 patients, middle thoracic esophagus in 63 patients, lower thoracic esophagus in 23 patients, and abdominal esophagus in 11 patients. The lesion was less than 50% circumferential in 96 patients and at least 50% circumferential in 15 patients. Triamcinolone acetonide (TA) was used to prevent stenosis in 26 patients. The histopathological diagnosis, based on the ESD/EMR guidelines for esophageal cancer [4], was dysplasia in 13 patients, squamous cell carcinoma (SCC) in 91 patients, and Barrett’s esophageal adenocarcinoma (BEA) in 7 patients. The pathological invasion depth of the SCC lesions was T1a-epithelial (EP) cancer in 45 patients, T1a-lamina propria mucosa (LPM) cancer in 32 patients, T1a-muscularis mucosae (MM) cancer in 7 patients, and T1b-submucosa (SM) cancer in 7 patients. The pathological invasion depth of the BEA lesions was T1a-epithelial (EP) cancer in 0 patients, T1a-superficial muscularis mucosae (SMM) cancer in 3 patients, T1a-lamina propria mucosa (LPM) cancer in 1 patient, T1a-deep muscularis mucosae (DMM) cancer in 0 patients, and T1b-submucosa (SM) cancer in 3 patients. The median operating time was 30 min (range 5–94 min) overall, 30 min (range 8–94 min) for experts, and 41 min (range 5–88 min) for trainees. Of the 111 esophageal ESD procedures, 78 were conducted by experts and 33 by trainees. The ESD perforation rate was 0% for both experts and trainees. The complete resection rate was 100% (Table 3).

## 3. Training for Safe, Efficient ESD

### 3.1. ESD Training Procedure

**Figure 1:**
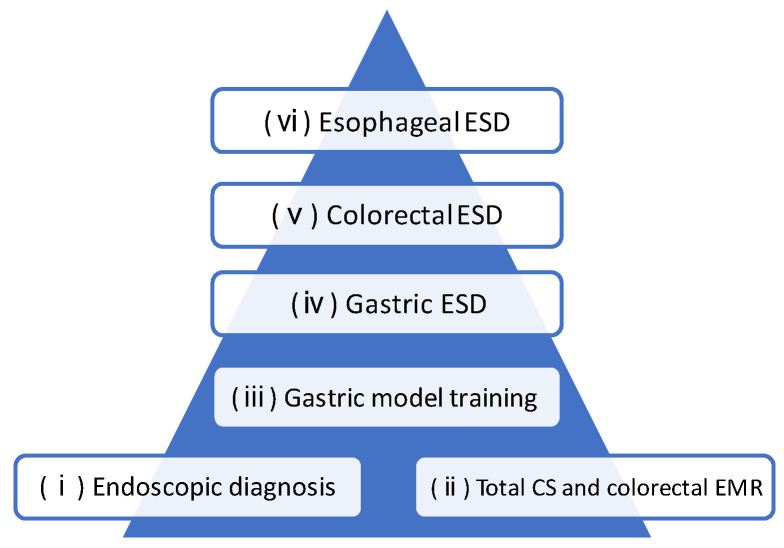
ESD training steps in our facility. Every step is supervised by experts. Abbreviations: ESD: endoscopic submucosal dissection; CS: colonoscopy; EMR: endoscopic mucosal resection.

The following steps must be mastered by trainees prior to performing ESD: (1) endoscopic diagnosis (the ability to correctly diagnose the invasion depth and extent of the ESD-indicated lesions using white light and magnified observations), (2) total colonoscopy and colorectal EMR (the ability to achieve cecum using the straightening and shortening method without stretching the intestinal tract and to perform EMR for basic colorectal tumorous lesions), and (3) gastric model training (the ability to skillfully create a mucosal flap for burrowing into the submucosal layer under the lesion). After these steps are completed, trainees are permitted to conduct ESD under complete one-to-one guidance by an expert, with training proceeding in the order of (4) gastric ESD, (5) colorectal ESD, and (6) esophageal ESD. The ESD treatment video was reviewed by the trainee and the supervising expert on the same day as each procedure to improve the trainee’s understanding of the details that they may not have noticed during the procedure (Figure 1). Trainees who are novices at ESD must conduct this procedure using safe methods. The perforation rate during ESD is believed to be associated with the number of procedures performed and is higher when less than 100 procedures have been completed [6]. The efficient conduct of the ESD procedure is also important to reduce the burden on patients and the risk of complications. 

### 3.2. Endoscopic Diagnosis

Previous studies have reported ESD training methods. Accurate preoperative diagnosis is a minimum requirement for the treatment of lesions via ESD. Recent research has focused on the use of artificial intelligence for endoscopic diagnoses of esophageal, gastric, and colorectal tumors [7,8,9]. However, endoscopists remain essential providers of endoscopic diagnoses. At our hospital, the endoscopic diagnoses determined by trainees must be checked by an expert. The expert confirms that the location and nature of the esophageal, gastric, or colorectal tumors and the preoperative diagnosis have been recorded appropriately and provides individual feedback to the trainee as needed. When an endoscopy is conducted for the purpose of further investigation, experts provide one-to-one guidance to trainees. Weekly ESD case feedback conferences and diagnostic endoscopy study meetings are also held at our hospital. The ESD conferences confirm the post-treatment pathology results and require that all of the participants review the histopathological presentations together. Comparing the endoscopic and pathological presentations allows participants to increase their knowledge and improve their endoscopic diagnosis abilities, including image-enhanced and magnified endoscopic observation. During the study meetings, the trainees interpret the endoscopic findings using pictures of endoscopic examinations previously conducted at our hospital. Then, an expert interprets the same pictures to educate the trainees regarding the important points in the examination, which the trainees summarize in a notebook for subsequent revision. The experts also assess whether the trainees have the minimum diagnostic skills required to perform the ESD procedure. 

### 3.3. Total Colonoscopy and Colorectal EMR

Colonoscopy is an important tool for the detection, diagnosis, and treatment of cancer, and learning the insertion method is a prerequisite for performing this procedure. When conducting colorectal ESD, the scope and the endoscopy screen must move as a unit with no flexure to manipulate the scope. The colorectum has five fixed points (the rectum, descending-sigmoid flexure, splenic flexure, hepatic flexure, and cecum), and the straight line joining these five points is the shortest route from the rectum to the cecum. The straightening and shortening method involves advancing the scope with the axis of the endoscope (the scope axis) aligned with the axis of the colorectum (the colorectal axis) and shortening the colorectum by folding its folds on top of one other [10]. At our hospital, this insertion method is used during colonoscopy and taught to each trainee by an expert. 

The ability to conduct EMR for basic tumorous lesions in the large intestine is another essential skill of endoscopists. Colorectal ESD requires a high level of proficiency and entails a greater risk of perforation than EMR [11,12,13,14,15]. Proper scope control is required during both EMR and ESD to maintain the stability and safety of the field of view. EMR may also be a useful procedure for supplementing total resection when en bloc resection via ESD is considered too technically difficult or entails too great a risk of perforation [16,17].

### 3.4. Gastric Model Training

Trainees who have mastered basic endoscopic diagnoses and the straightening and shorting insertion method and those who have been assessed by an expert as capable of conducting colorectal EMR advance to gastric model training prior to conducting the ESD procedure. In the past, animal models were typically used for gastric ESD training [18,19]. Vázquez-Sequeiros et al. reported that after four endoscopists with no ESD experience had undergone ex vivo and in vivo training using an animal model, they were able to conduct ESD in human patients with no complications [18]. Parra-Blanco et al. also reported that when endoscopists with no clinical ESD experience underwent ex vivo and in vivo gastric model training on a total of 21 pigs, the mean resection time was significantly shorter during the second part of the training (8 pigs) than during the first part of training (13 pigs) [19]. However, the stomach has anatomical characteristics that are not completely replicated by animal models. In addition, as gastric cancer lesions may be located in several different sites within the stomach, the level of difficulty of ESD varies greatly, requiring a skilled endoscopist [20,21,22,23]. There are also hygienic and cost concerns regarding animal models. The COVID-19 pandemic has raised the public’s awareness regarding infectious diseases, making it more difficult to use model animals in a clinical endoscopy room. This is a major challenge for trainees. Therefore, a new gastric ESD training model, the G-Master, has been developed in a joint research project between KOTOBUKI Medical Limited and the National Cancer Center Hospital East. In this model, lesions in 11 specific locations within the stomach can be replicated. The use of G-Master in conjunction with versatile training tissue (VTT; KOTOBUKI Medical, Saitama, Japan), a plant-based sheet consisting primarily of konjac powder, developed originally for laryngopharyngeal cancer surgery training, allows for more realistic training scenarios.

**Figure 2:**
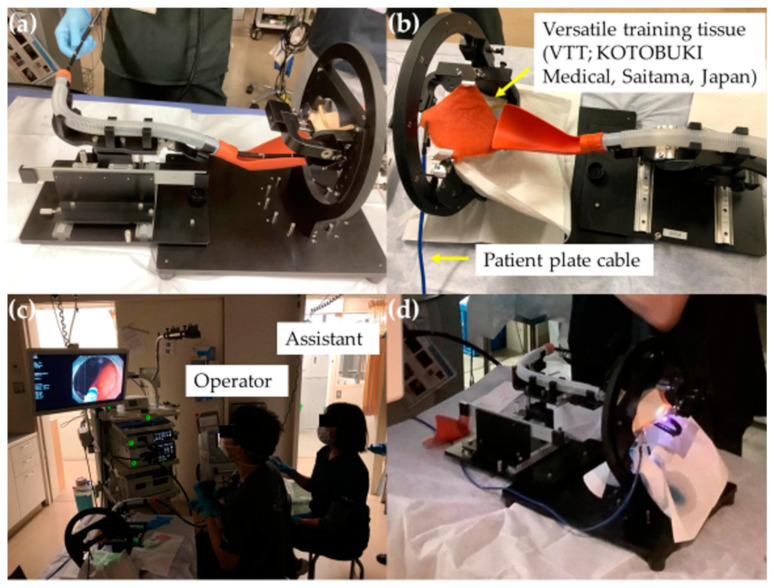
(**a**,**b**) G-Master; (**c**,**d**) training scenery using the G-Master.

**Figure 3:**
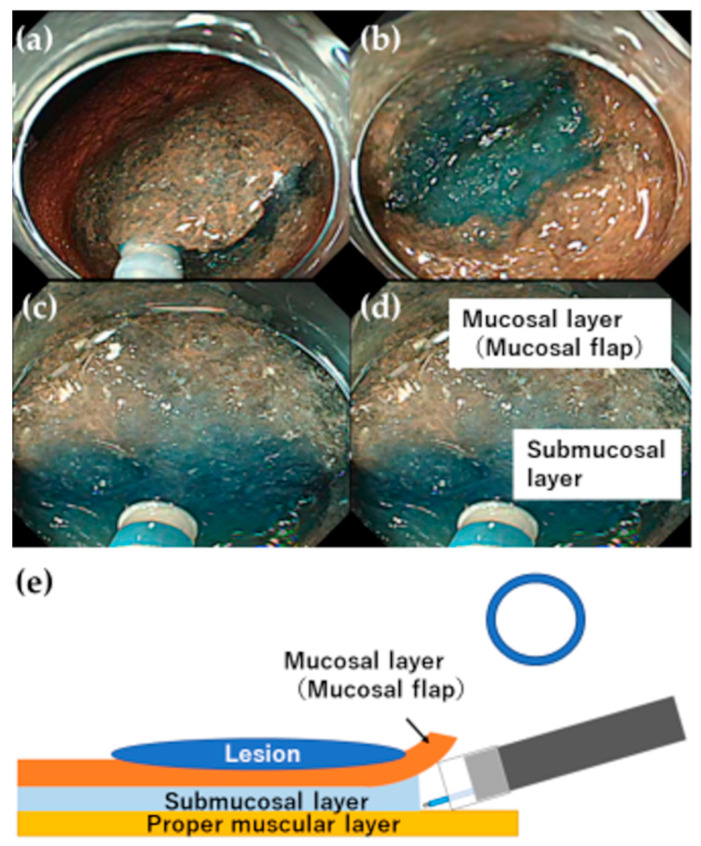
Making a mucosal flap in G-Master training. (**a**) While there is sufficient localized fluid injected to the submucosal layer, a short distance mucosal incision and submucosal dissection (trimming) of the area should be repeated. (**b**) After trimming several times, the distal attachment can be used to burrow under the submucosal layer (success in making the mucosal flap). (**c**) While keeping the visual field, dissect the submucosal layer so that it is parallel to the muscular layer. (**d**) Description of the layers. (**e**) Schema diagram.

**Figure 4:**
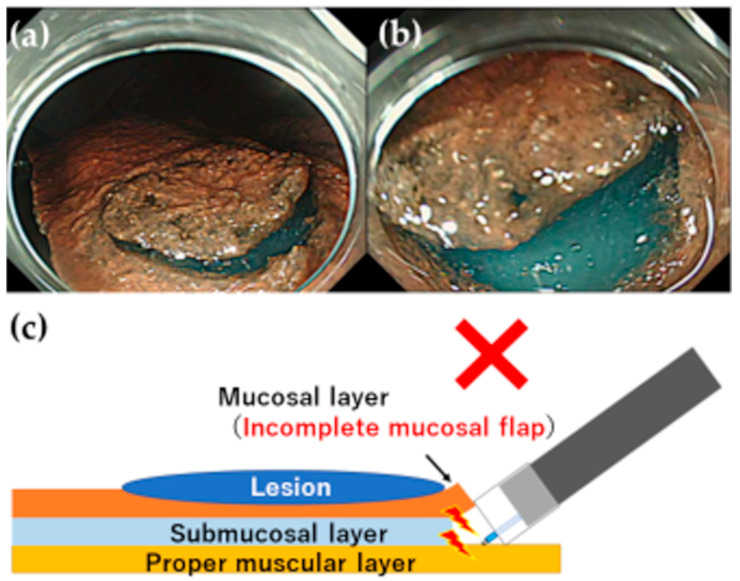
Incomplete mucosal flap in G-Master training. (**a**) If the mucosal incision is extended too wide with inadequate trimming, it is difficult to obtain sufficient submucosal swelling because the injected fluid is spread over a wide area. (**b**) Because no space can be created between the mucosal and submucosal layers, the distal attachment cannot burrow under the submucosal layer (failure to make the mucosal flap). (**c**) Schema diagram.

Mitsui et al. evaluated the actual resection speed using G-Master and its ease of use, and reported that it is useful for gastric ESD training [24]. G-Master is used in our department for training prior to the conduct of gastric ESD procedures (Figure 2). We consider model training using G-Master as an important session to improve technique before and even after starting patient ESD. Therefore, model training sessions are generally conducted under the guidance of an expert. We have also created a VTT training model simulating the gastrointestinal tract using a plastic apron box, gauze, and rods, which are readily available in the endoscopy room, providing basic ESD training at a low cost [25]. A local injection of hyaluronic acid during VTT results in a substitute material for the gastric mucosa that can be used to practice making an incision with an ESD knife. The speed with which a mucosal flap for burrowing under the submucosal layer can be produced limits all types of ESD procedure. If a mucosal flap can be created, the incision can be safely and efficiently extended to proceed with the treatment. However, if the operator detaches the mucosa and cannot burrow under the submucosal layer as the lesion remains attached to the submucosa, they will be cutting blindly, increasing the risk of perforation and the time required for hemostasis. Therefore, our trainees must be able to efficiently create a mucosal flap using the gastric model prior to conducting an ESD procedure on a patient (Figure 3 and Figure 4 and Video S1).

### 3.5. Gastric ESD

#### 3.5.1. ESD Devices and Settings

Gastric ESD is conducted with the patient under conscious sedation, using midazolam and pethidine hydrochloride. A GIF-H290Z endoscope (Olympus Medical Systems Corp., Tokyo, Japan), a GIF-H290T endoscope (Olympus Medical Systems), and a GIF-Q260J endoscope (Olympus Medical Systems) are typically used. When it is difficult to bring the scope close to the fornix or the annulus, a GIF-2TQ260M endoscope (Olympus Medical Systems), which is a multi-bending two-channel scope, is used. The D201-11804 distal attachment (Olympus Medical Systems) is used with the GIF-H290Z, the GIF-H290T, and the GIF-Q260J endoscopes, and a D-201-14304 distal attachment (Olympus Medical Systems) is used with the GIF-2TQ260M endoscope. MucoUP (Boston Scientific, Tokyo, Japan), a sodium hyaluronate solution, is injected locally into the submucosal layer. A dual knife J (KD-655L; Olympus Medical Systems), with an incision knife length of 2.0 mm, is used as the basic ESD knife. To improve the efficiency of the dissection, when the mucosal layer is in the frontal view and the use of the needle-type knife is challenging, or for severely fibrotic lesions, an IT knife nano (KD-612L; Olympus Medical Systems), a mucosectom (DP-D2518/DP-D2622; PENTAX Medical, Tokyo, Japan), or a ClutchCutter (DP2618DT-35/DP2618DT-50-; Fujifilm Medical, Tokyo, Japan) is used as a second device, depending on the patient. A high-frequency electrosurgical unit (VIO 3; Erbe Elektromedizin, Tübingen, Germany) is used for all procedures. Mucosal incision and submucosal dissection are conducted using the endocut mode (endocut I, effect 1, duration 4, and interval 1) and the coagulation mode (forced coagulation and effect 7.0). If bleeding occurs, the coagulation mode (spray coagulation and effect 1.5) is used with the dual knife J tip closed. If bleeding occurs with another device or the bleeding is from a large vessel and hemostasis cannot be achieved via spray coagulation, a Coagrasper (FD-410LR; Olympus Medical Systems), the hemostatic forceps with an opening width of 5 mm, are used in coagulation mode (soft coagulation and effect 3.0).

#### 3.5.2. Training and Conduct of Safe, Efficient ESD

Trainees who successfully complete gastric model training on G-Master are permitted to conduct an ESD procedure on a patient, with one-on-one guidance provided by an expert. Oda et al. [26] and Yoshida et al. [27] have reported that at least 30 procedures are required to improve one’s gastric ESD skills and that novices should start with ESD on the lower third of the stomach, which is less difficult than other gastric ESD procedures. At our hospital, trainees begin performing ESD in the lower part of the stomach, such as the antrum. Rather than completing an entire procedure, the trainees begin by efficiently creating a mucosal flap, as practiced during G-Master training. Once they are able to create this mucosal flap within a few minutes, their training proceeds toward completing the entire procedure. The management of intraoperative hemostasis is an important aspect of safe, efficient gastric ESD. If the amount of bleeding is large and the source is difficult to identify, then hemostasis must be achieved using the ESD knife or hemostatic forceps. The resulting carbonization of the submucosal tissue may interfere with the field of view or make the line of dissection difficult to visualize, increasing the risk of perforation [28,29,30]. The burden on the patient is increased if a large volume of irrigation water is required to identify the hemostasis point, or the amount of air in the stomach increases. The patient may move or burp, increasing the difficulty of the ESD procedure. Therefore, the proper management of intraoperative hemorrhage and the accurate identification of the source of bleeding are important for the success of ESD procedures. According to Oyama et al., the most important method to prevent intraoperative bleeding during ESD is the identification of blood vessels in the submucosa and the application of prophylactic coagulation hemostasis before these vessels are cut [31]. Toyonaga et al. reported that identifying the true submucosal layer, which has few blood vessels and contains residual fibrotic tissue, is important for reducing intraoperative bleeding during ESD [32]. At our hospital, long coagulation waves are used to provide heat coagulation for hemostasis when blood vessels in the submucosa are identified, and hemostatic forceps are used for the coagulation of large arterial vessels. However, it is sometimes difficult to identify vessels before they bleed, and the accidental cutting of a vessel is sometimes unavoidable. Trainees tend to apply coagulation waves frequently while the field of view remains narrow to stop bleeding quickly without proceeding with mucosal incision or dissection. Until the bleeding site has been accurately identified, hemostasis is typically not effective, and the submucosa is carbonized by the coagulation heat, resulting in its contraction, which renders the creation of a mucosal flap impossible. In addition, if the proper incision line can no longer be identified due to the carbonization of the tissue, the risk of perforation increases. Therefore, if bleeding occurs during the mucosal incision or submucosal dissection, it is important to continue detaching the mucosa or submucosa to open up the field of view. Extending the field of view makes it easier to identify the bleeding vessel, so that proper hemostasis be performed. Reducing the time required for hemostasis also reduces the overall operating time for ESD. Maehata et al. reported that the new image-enhancement technique for dual red imaging is useful for identifying the hemostasis point, when intraoperative bleeding occurs during ESD [33].

Gel immersion endoscopy has been developed with the aim of securing the field of view in the event of gastrointestinal hemorrhage, by utilizing the high viscosity of gel [34]. An electrolyte-free gel (Viscoclear; Otsuka Pharmaceutical Factory, Inc., Tokushima, Japan) exerts an effective anticoagulant capacity within the gel [35]. Khurelbaatar et al. reported that this electrolyte-free gel is useful for identifying the source of intraoperative bleeding during gastric ESD and that coagulation hemostasis using the gel was effective [36]. The further development of new procedures and techniques will allow for the improvement of the safety and efficacy of ESD.

### 3.6. Colorectal ESD

#### 3.6.1. ESD Devices and Settings

Colorectal ESD is also conducted in the endoscopy room with the patient under conscious sedation with midazolam and pethidine hydrochloride. For lesions in the colon, the PCF-H290ZI endoscope (Olympus Medical Systems) and the PCF-H290TI endoscope (Olympus Medical Systems) are typically used. For rectal lesions, the GIF-H290T endoscope (Olympus Medical Systems), which is a general-use upper gastrointestinal scope, is often used, as it is easier to manipulate with a shorter effective length and the down angle is more effective. A D-201-12704 distal attachment (Olympus Medical Systems) is used with the PCF-H290ZI endoscope, a D-201-11304 distal attachment (Olympus Medical Systems) is used with the PCF-H290T endoscope, and a D-201-11804 distal attachment (Olympus Medical Systems) is used with the GIF-H290T endoscope. MucoUP (Boston Scientific, Tokyo, Japan), a sodium hyaluronate solution, is locally injected into the submucosal layer. A dual knife J (KD-650Q; Olympus Medical Systems) with an incision knife length of 1.5 mm is used as the basic ESD knife. To improve the efficiency of the dissection, either an IT knife nano (KD-612Q; Olympus Medical Systems), a mucosectom (DP-D2622; PENTAX Medical, Tokyo, Japan), or a ClutchCutter (DP2618DT-35-; Fujifilm Medical, Tokyo, Japan) is used when the mucosal layer is in the frontal view and the needle-type knife cannot be easily used, or the lesion is severely fibrotic. Mucosal incision and submucosal dissection are conducted in the endocut mode (endocut I, effect 1, duration 4, and interval 1) and the coagulation mode (forced coagulation and effect 6.0). If bleeding occurs when the dual knife J is used, the coagulation mode (spray coagulation and effect 1.5) is used with the knife tip closed. If another device is in use or the bleeding is from a large vessel, so that hemostasis cannot be achieved using the spray coagulation mode, a Coagrasper (FD-411QLR; Olympus Medical Systems), the hemostatic forceps with an opening width of 4 mm, are used in coagulation mode (soft coagulation and effect 3.0).

#### 3.6.2. Training and Conduct of Safe, Efficient ESD

Trainees who can perform a basic gastric ESD next learn to perform a colorectal ESD. It is recommended that colorectal ESD is first conducted on laterally spreading, granular-type lesions measuring 2–3 cm in the rectum, as these tumors have a low risk of submucosal invasion, and the scope of operability is typically better in the rectum than in the colon [37]. According to Hotta et al., approximately 40 procedures must be performed to acquire sufficient ESD skills to conduct the procedure without perforation, and the perforation rate during these first 40 procedures is expected to be approximately 12.5% [38]. Sakamoto et al. reported that 30 colorectal ESD procedures are necessary to acquire the skills required to complete ESD independently [39]. Although it is thought that 30–40 procedures are sufficient for the improvement of trainees’ skills, the increased risk of perforation is a concern. Perforations are associated with physical adverse effects for the patient, as well as being a major psychological burden both for the trainee and the supervising expert who conducted the procedure. In our department, experts and trainees have a perforation rate of 0%. 

**Figure 5:**
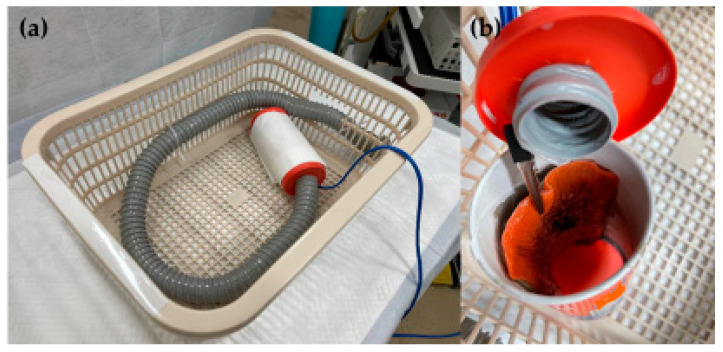
(**a**) A colorectal training model made from VTT, a basket, an accordion hose, and a tubular snack box. (**b**) The snack box contains a VTT with the electrode attached. Abbreviations: VTT: versatile training tissue.

Few previous studies have suggested that performing safe ESD procedures during training may require the use of animal models. Hon et al. developed a training model using a resected porcine distal colon that costs USD 30. The operating time gradually decreased after trainees performed 10 ESD procedures using this animal model [40]. Iacopini et al. studied a short-term, intensive, stepwise training method using an artificial training model made from a resected porcine stomach, in combination with supervision and intervention by an ESD expert. Trainee endoscopists exhibited a good learning curve when subsequently conducting ESD on the rectums and colons of human patients, with the operating time decreasing in accordance with the number of procedures performed [41]. A colorectal training model made from VTT, a basket, an accordion hose, and a tubular snack box that enables hygienic training at a lower cost than that of animal models has also been reported. This model has the distinctive feature that, in addition to the forward view, training in retroflex view, which is necessary for some lesions, is also feasible [42] (Figure 5). In our department, we also incorporate model training while allowing trainees to begin conducting ESD procedures. Once the trainee has acquired the necessary skills, he or she can conduct colonic ESDs outside the rectum.

The goal of colorectal ESD is for the trainee to continue training thoroughly until he or she can efficiently create the mucosal flap required to burrow under the submucosal layer directly beneath the lesion. The creation of sufficient space for the scope to burrow under the submucosa is the key to success in colorectal ESD. Therefore, limiting the width of the initial mucosal incision and trimming, via repetitive submucosal dissection directly beneath the lesion, is effective [43].

Trainees who are able to create the mucosal flap advance to completing the procedure in a human patient. The traction method is used to train endoscopists to conduct the procedure efficiently. During the traction method, a device is used to apply counter traction to the lesion and secure a good field of view while it is resected. Sakamoto et al. reported a safe and efficient traction technique using an SO clip (TC1H05; Zeon Medical Co., Ltd., Tokyo, Japan) [44,45]. Fujinami et al. compared the treatment time, incision speed, en bloc resection rate, and perforation rate of colorectal ESD conducted using the conventional method without an SO clip with the rates when an SO clip was used and found that the SO clip method results in significantly shorter treatment times, significantly faster resection speeds, significantly higher en bloc resection rates, and significantly lower perforation rates [46]. In our department, we proactively use an SO clip when the lesion is large or expected to be severely fibrotic, and when its use may allow for a shorter operating time and more efficient treatment. 

### 3.7. Esophageal ESD

#### 3.7.1. ESD Setting

Esophageal ESD is conducted in the endoscopy room with the patients under sedation with dexmedetomidine, midazolam, and pethidine hydrochloride. Many esophageal patients have consumed large amounts of alcohol, leading to midazolam being less effective. However, increasing the dose of midazolam may cause respiratory depression or induce rough body movements due to disinhibition. Therefore, dexmedetomidine is also used so that the dose of midazolam can be reduced [47,48,49].

The basic endoscope used during esophageal ESD is the GIF-H290T endoscope (Olympus Medical Systems) or the GIF-Q260J endoscope (Olympus Medical Systems). When the extent of the lesion is difficult to diagnose, such as in patients with Barrett’s esophageal carcinoma or patients with an iodine allergy for whom Lugol’s iodine spray cannot be used, a magnifying endoscope, such as the GIF-H290Z endoscope (Olympus Medical Systems), is used. The D201-11804 distal attachment (Olympus Medical Systems) is used with the GIF-H290T, the GIF-Q260J, and the GIF-H290Z endoscopes. MucoUP (Boston Scientific, Tokyo, Japan) is locally injected into the submucosal layer. A dual knife J (KD-650QL; Olympus Medical Systems) with an incision knife length of 1.5 mm is used as the basic ESD knife. To improve the efficiency of the dissection, when the mucosal layer is in the frontal view and the needle-type knife cannot easily be used or for severely fibrotic lesions, an IT knife nano (KD-612L; Olympus Medical Systems), a mucosectom (DP-D2622; PENTAX Medical, Tokyo, Japan), or a ClutchCutter (DP2618DT-35-; Fujifilm Medical, Tokyo, Japan) is used as a second device, depending on the patient. A high-frequency electrosurgical unit (VIO 3; Erbe Elektromedizin, Tübingen, Germany) is used for all patients, and mucosal incision and submucosal dissection are carried out in the endocut mode (endocut I, effect 1, duration 4, and interval 1) and the coagulation mode (forced coagulation and effect 6.0). If bleeding occurs and the dual knife J is used, the coagulation mode (spray coagulation and effect 1.5) is used with the knife tip closed. If another device is used or the bleeding is from a large vessel and hemostasis cannot be achieved via spray coagulation, a Coagrasper (FD-411QR; Olympus Medical Systems), the hemostatic forceps with an opening width of 4 mm, are used in coagulation mode (soft coagulation and effect 3.0).

#### 3.7.2. Training and Conduct of Safe, Efficient ESD

The muscularis propria of the esophagus is thinner than that of the stomach and has no serosa, increasing the risks of perforation and pneumomediastinum. A study using the Japanese nationwide administrative database reported that the perforation rate during esophageal ESD is approximately 3.3% [50]. The esophagus has a narrow lumen and is affected by displacement of the surrounding organs. Therefore, it may be difficult to obtain an appropriate field of view depending on the location of the lesion. The esophagus is also susceptible to the effects of pulsation and respiratory movements, making endoscopic manipulation difficult. Therefore, esophageal ESD is extremely difficult. In our department, training is provided under expert instruction at an early stage if the trainee has acquired sufficient experience and skills to complete gastric and colorectal ESD procedures. However, due to the high degree of difficulty of esophageal ESD and the potentially fatal complications associated with the procedure, esophageal ESD is mainly conducted by experts. Only trainees who can successfully perform gastric and colorectal ESD procedures and have been assessed by an expert as capable of conducting safe, reliable ESD procedures are permitted to train for esophageal ESD.

**Figure 6:**
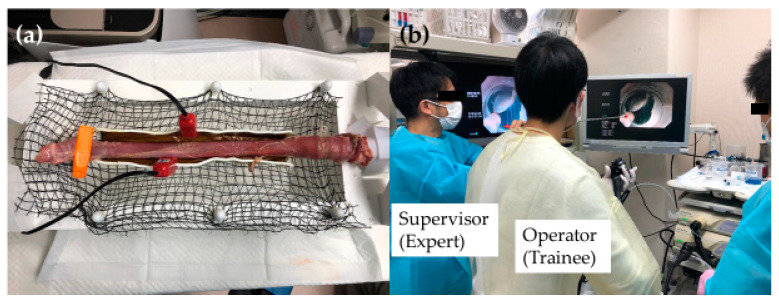
Esophageal ESD training using an ex vivo porcine model. (**a**) An ex vivo porcine model; (**b**) a training scene showing the instruction of an expert. Abbreviations: ESD: endoscopic mucosal dissection.

**Figure 7:**
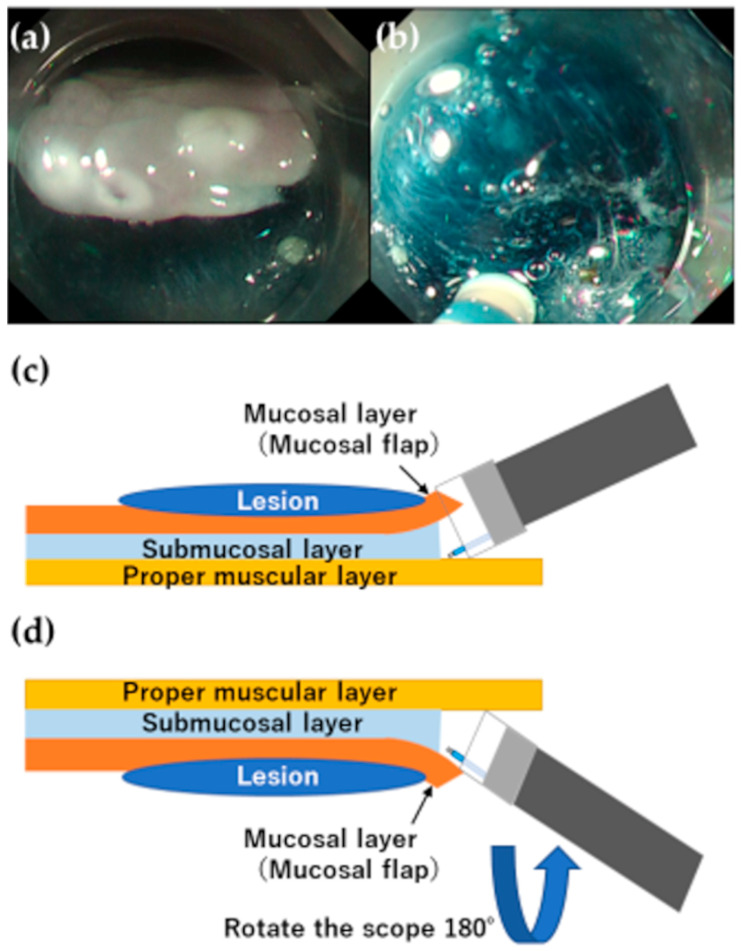
(**a**) Normal position (the esophageal wall at the 6 o’clock position for submucosal dissection). In this case, the flap is more likely to enter the distal attachment when the device is approached toward the submucosal layer. Furthermore, the device that comes out from the underside, the 6 o’clock direction of the scope, is close to the muscular layer, so there is a risk of damaging the muscular layer. (**b**) The “upside-down” position (the esophageal wall at 12 o’clock for submucosal dissection). By rotating the scope 180°, the device that comes out from the underside, allows the dissection to be made away from the muscular layer. The risk of perforation can be minimized as much as possible by recognizing the safe direction of the esophageal lumen where the device should move and the dissection. (**c**) Schema diagram of the normal position. (**d**) Schema diagram of the “upside-down” position.

Few studies have described the use of animal models for esophageal ESD training. Tanimoto et al. used an in vivo canine model to conduct esophageal ESD procedures and reported a good learning curve in terms of the perforation rate and resection time among postgraduate endoscopy fellows [51]. Tanaka et al. used ten ex vivo porcine esophagus models to train three endoscopists with experience conducting gastric ESD for esophageal ESD and compared the therapeutic outcomes for the first five and the second five models. They reported fewer sites of muscle damage and shorter ESD operating times in the second five models than in the first five models for all three endoscopists [52]. Therefore, esophageal ESD training with an animal model prior to conducting the procedure on human patients is important for achieving safer, more efficient treatment. In our department, we implement esophageal ESD training using an ex vivo porcine model (Figure 6). This model of training is useful for learning the basic esophageal ESD procedure, though pulsation and respiratory movements present additional challenges during actual procedures. An ex vivo porcine model with an attached electric motor that rotates at 80 cycles/min, a rate similar to that of the human pulse, has been reported [53]. This model allows trainees to have a more realistic experience during training. Trainees who have acquired the basic skills required for esophageal ESD via animal model training are permitted to continue training by conducting the procedure on human patients. Similar to the training for gastric and colorectal ESD, training for esophageal ESD starts with the creation of a mucosal flap on the oral side of the lesion. Once the trainee is able to create an oral-side mucosal incision and trim to create the mucosal flap, he or she uses the distal attachment to assess the space to burrow under the submucosal layer. If there is sufficient space for burrowing, the trainee proceeds to submucosal dissection, avoiding damage to the muscle layer by rotating the scope so that the esophageal wall into which the incisions are to be made (the region where detachment is to be conducted) is at the 12 o’clock position for dissection, termed the “upside-down” position (Figure 7). This angle allows for the incisions to be made at a greater distance from the muscle layer when the device that comes out from the underside of the scope is close to the 6 o’clock position. If incision and dissection are conducted with an awareness of the direction in which the device in the esophageal lumen can be safely withdrawn, the risk of perforation is minimized. If the scope sticks too close to the submucosal layer and the device is perpendicular to the muscle layer, the risk of perforation is high. Therefore, incisions must be made with the device at an angle that is approximately parallel to the muscle layer and the device should be extended further than usual to maintain an appropriate distance between the scope and the tissue to be cut. 

Counter traction can be used to open up the field of view, allowing for safe and efficient esophageal ESD. Oyama et al. described the clip with line method for safe and efficient dissection by grasping the oral end of the lesion with a clip to which a line is attached and pulling the line orally to achieve good traction, while burrowing under the submucosal layer with the scope [54]. The clip with line method is used on most patients at our hospital. We also often use an IT knife nano (KD-612; Olympus Medical Systems) as a second device. The IT knife nano has a short length and a small, insulated tip, making it suitable for ESDs in organs, such as the esophagus and colorectum, where the submucosal space is narrow [55]. Because the tip of this device is insulated, the risk of mistakenly damaging the muscle layer is minimalized, and the small size of the device enables safe submucosal dissection in the forward direction under direct observation. Kitagawa et al. have reported that the combined use of the clip with line method and the IT knife nano is useful for achieving safe, efficient esophageal ESD [56]. Therefore, esophageal ESD requires additional methods to ensure its safety, when compared to gastric and colorectal ESD. However, longer operation times increase the patient burden, and patient movement increases the difficulty of the procedure and the risk of complications. Therefore, efficient procedures are necessary.

#### 3.7.3. Prevention of Post-Esophageal ESD Stenosis

Post-esophageal ESD stenosis may occur even after a safe and efficient procedure. The current advances in esophageal ESD technology allow for extensive lesions, including subcircumferential and circumferential lesions, to be removed en bloc. However, the rate of postoperative stenosis after extensive esophageal ESD is high. Patients with cervical esophageal lesions and those who have undergone radiotherapy also have a high risk of postoperative stenosis and require careful management. Dilation is often necessary if stenosis occurs, which significantly affects the patient’s quality of life. Therefore, appropriate methods should be used to prevent stenosis in high-risk patients.

After esophageal ESD, myofibroblasts appear above the thinned muscle layer of the esophagus, resulting in a thickened layer. Myofibroblasts are spindle-shaped, contractile cells that are arranged in parallel [57]. In animal experiments, myofibroblast proliferation results in post-ESD stenosis and a similar mechanism may also cause stenosis in humans [58]. Local steroid injections reduced the number of myofibroblasts and caused morphological changes to the cells in animal experiments [57]. These changes may decrease the contractility of the myofibroblasts and alleviate esophageal stenosis. Methods of preventing stenosis include local steroid injections [59], oral steroids [60], prophylactic balloon dilatation [61], and the application of a polyglycolic acid sheet [62], or an oral mucosal epithelial cell sheet [63]. In real-world clinical practice, a combination of prophylactic balloon dilatation and either local steroid injection or oral steroids is typically used. At our hospital, we have devised an unusual approach to local steroid injection. Local steroid injection is a method of injecting TA into the remaining thin submucosal layer after ESD [59]. In general, TA is injected into the submucosal layer using a local injection syringe, though this thin, sharp needle may pass through the submucosal layer, resulting in the injection of TA into the muscle layer. One study reported that the accidental injection of TA into the muscle layer increases the risk of esophageal perforation and abscess formation [64]. Therefore, at our hospital, TA is dissolved in physiological saline at a concentration of 5 mg/mL, and the injection needle is kept closed in the sheath and injected in small amounts as if stamping on the remaining submucosal layer. This technique prevents the accidental injection of TA into the muscle layer and potential perforation, resulting in a safe, uniform local injection. The use of a spray tube has also been reported [65]. 

In our department, trainees learn how to provide treatment in consideration of the patients’ quality of life during and after the procedure. Most trainees who study in our department are doctors from other Japanese hospitals or doctors from other university hospitals who are participating in a study exchange. After training in our department, the physicians often return to their own institutions to apply their knowledge and treat patients at those hospitals. Therefore, we have designed a system under which trainees are taught how to conduct the entire ESD procedure from diagnosis to treatment and postoperative management.

## 4. Conclusions

Recent improvements in endoscopists’ skills and technological advances have allowed ESD to become a standard procedure for gastrointestinal tumors that can be conducted in general hospitals. Therefore, general endoscopists must be trained to provide safe, efficient, and accurate treatment. We have been conducting safe ESD with a perforation rate of 0% among trainees and experts at our hospital. Planned, one-to-one training of ESD trainees is important for maintaining this perforation rate.

## Figures and Tables

**Table 1:** Clinicopathological data of patients who underwent gastric ESD.

Clinicopathological Data	N = 346
Age (years), median (range)	73 (35–92)
Sex, no. (%)	
Male	252 (72.8)
female	94 (27.2)
Median lesion size, mm (range)	
Overall	13.5 (1–100)
Expert	14 (1–100)
Trainee	8 (1–68)
Tumor subsites, no. (%)	
Upper third	63 (18.2)
Middle third	109 (31.5)
Lower third	174 (50.3)
Histopathological diagnosis, no. (%)	
Adenoma	53 (15.3)
Carcinoma	274 (79.2)
Differentiated type	239 (87.2)
Undifferentiated type	35 (12.8)
Others	19 (5.5)
Depth of invasion, no. (%)	
Intramucosal (T1a) carcinoma	232 (67.1)
Submucosal invasive (T1b) carcinoma	41 (32.9)
Median operating time, min (range)	
Overall	35.5 (3–277)
Expert	30 (3–277)
Trainee	38 (3–190)
Operator, no. (%)	
Expert	137 (39.6)
Trainee	209 (60.4)
Perforation no. (%)	
Expert	0 (0)
Trainee	0 (0)
Complete resection (R0), no. (%)	341 (98.6)

Data are presented as median (range) or number (percentage). Abbreviation: ESD: endoscopic submucosal dissection.

**Table 2:** Clinicopathological data of patients who underwent colorectal ESD.

Clinicopathological Data	N = 361
Age (years), median (range)	68 (32–91)
Sex, no. (%)	
Male	208 (57.6)
Female	153 (42.4)
Median tumor size, mm (range)	
Overall	23 (4–88)
Expert	26 (5–88)
Trainee	20 (4–67)
Tumor subsites, no. (%)	
Colon	268 (74.2)
Rectum	93 (25.8)
Histopathological diagnosis, no. (%)	
Adenoma	99 (27.4)
Intramucosal (Tis) carcinoma	149 (41.3)
Submucosal invasive (T1) carcinoma	39 (10.8)
Sessile serrated lesion	67 (18.6)
Others	7 (1.9)
Median operating time, min (range)	
Overall	37 (5–294)
Expert	36 (5–294)
Trainee	38 (8–118)
Operator, no. (%)	
Expert	197 (54.6)
Trainee	164 (45.4)
Perforation no. (%)	
Expert	0 (0)
Trainee	0 (0)
Complete resection (R0), no. (%)	357 (98.9)

Data are presented as median (range) or number (percentage). Abbreviation: ESD: endoscopic submucosal dissection.

**Table 3:** Clinicopathological data of patients who underwent esophageal ESD.

Clinicopathological Data	N = 111
Age (years), median (range)	71 (50–87)
Sex, no. (%)	
Male	92 (82.9)
Female	19 (17.1)
Median tumor size, mm (range)	
Overall	16 (4–61)
Expert	18 (4–61)
Trainee	15 (5–41)
Tumor subsites, no. (%)	
Cervical esophagus	2 (1.8)
Upper thoracic esophagus	12 (10.8)
Middle thoracic esophagus	63 (56.7)
Lower thoracic esophagus	23 (20.7)
Abdominal esophagus	11 (10.0)
Circumference of the lesion, no. (%)	
<1/2	96 (86.5)
≥1/2	15 (13.5)
TA injection after ESD, no. (%)	26 (23.4)
Histopathological type, no. (%)	
Dysplasia	13 (11.7)
SCC	91 (82.0)
BEA	7 (6.3)
Depth of invasion, no. (%)	
SCC	
T1a-EP cancer	45 (49.5)
T1a-LPM cancer	32 (35.1)
T1a-MM cancer	7 (7.7)
T1b-SM cancer	7 (7.7)
BEA	
T1a-EP cancer	0 (0)
T1a-SMM cancer	3 (42.9)
T1a-LPM cancer	1 (14.2)
T1a-DMM cancer	0 (0)
T1b-SM cancer	3 (42.9)
Median operating time, min (range)	
Overall	30 (5–94)
Expert	30 (8–94)
Trainee	41 (5–88)
Operator, no. (%)	
Expert	78 (70.3)
Trainee	33 (29.7)
Perforation no. (%)	
Expert	0 (0)
Trainee	0 (0)
Complete resection (R0), no. (%)	111 (100)

Data are presented as median (range) or number (percentage). Abbreviations: TA: triamcinolone acetonide; ESD: endoscopic submucosal dissection; SCC: squamous cell carcinoma; BEA: Barrett’s esophageal adenocarcinoma; EP: epithelial; LPM: lamina propria mucosa; MM: muscularis mucosae; SM: submucosa; SMM: superficial muscularis mucosae; DMM: deep muscularis mucosae.

## Data Availability

Not applicable.

## Supplementary Materials

The following supporting information can be downloaded at: https://www.mdpi.com/article/10.3390/jcm12113692/s1, Video S1: Mucosal flap making in G-Master.
